# Sensitivity of Papilloma Virus-Associated Cell Lines to Photodynamic Therapy with Curcumin-Loaded Liposomes

**DOI:** 10.3390/cancers12113278

**Published:** 2020-11-05

**Authors:** Ghazala Ambreen, Lili Duse, Imran Tariq, Uzma Ali, Sajid Ali, Shashank R. Pinnapireddy, Michael Bette, Udo Bakowsky, Robert Mandic

**Affiliations:** 1 Department of Pharmaceutics and Biopharmaceutics, Philipps-Universität Marburg, 35037 Marburg, Germany; ghazala.ambreen@pharmazie.uni-marburg.de (G.A.); lili.duse@pharmazie.uni-marburg.de (L.D.); Imran.pharmacy@pu.edu.pk (I.T.); uzma.ali@pharmazie.uni-marburg.de (U.A.); Alisaj@staff.uni-marburg.de (S.A.); shashank.pinnapireddy@pharmazie.uni-marburg.de (S.R.P.); 2 Department of Otorhinolaryngology, Head and Neck Surgery, University Hospital Marburg, Philipps-Universität Marburg, 35033 Marburg, Germany; 3 Punjab University College of Pharmacy, University of the Punjab, Allama Iqbal Campus, 54000 Lahore, Pakistan; 4 Faculty of Pharmacy, The University of Lahore, 54000 Lahore, Pakistan; 5 CSL Behring GmbH, 35041 Marburg, Germany; 6 Institute of Anatomy and Cell Biology, Philipps-Universität Marburg, 35037 Marburg, Germany; bette@staff.uni-marburg.de

**Keywords:** head and neck squamous cell carcinoma (HNSCC), head and neck cancer, cervical cancer, photodynamic therapy, curcumin liposomes, papilloma virus

## Abstract

**Simple Summary:**

Globally, the burden of papilloma virus-associated cancers is high. About 5% of all cancers worldwide are caused by the human papillomavirus (HPV). Photodynamic therapy (PDT) is considered as a useful therapeutic option to treat cancers, particularly those near the tissue surface, since it is typically well tolerated and less invasive with a lower risk of severe complications as compared to conventional treatment strategies. PDT requires the combination of a photosensitizer, light of a specific wavelength, and tissue oxygen. In the present study, we examined the effectiveness of PDT together with a curcumin (liposome)-based photosensitizer in three papilloma virus-associated cell lines. PDT with curcumin liposomes could inhibit proliferation, cell migration, and colony formation of the tested tumor cells. The results suggest that curcumin-encapsulated liposomes in conjunction with PDT could be a useful tool for the treatment of papilloma virus-associated tumors.

**Abstract:**

Photodynamic therapy (PDT) is a minimally invasive therapeutic approach used in the treatment of various medical conditions and cancerous diseases, involving light, a photosensitizing substance, and oxygen. Curcumin, a naturally occurring compound, carries antitumor activities and potentially could be exploited as a photosensitizer in PDT. Only little is known about liposomal-encapsulated curcumin that could help in increasing the efficacy, stability, and bioavailability of this compound. This study investigates the in vitro effects of curcumin-loaded liposomes in combination with PDT. Three papilloma virus-associated cell lines were treated with curcumin-loaded liposomes corresponding to a curcumin concentration of 0–100 µmol/L for 4 h followed by illumination at 457 nm (blue) for 45, 136, and 227 s at a fluence of 220.2 W/m^2^ (100 mA) corresponding to 1, 3 and 5 J·cm^−2^. After 24 h, the biological outcome of the treatment was assessed with the MTT (3-(4,5-dimethylthiazol-2-yl)-2,5-diphenyltetrazolium bromide), SYTO9/PI (propidium iodide), Annexin V-FITC (fluorescein isothiocyanate)/PI, clonogenic survival, and scratch (wound closure) assays. Photoactivation of curcumin-loaded liposomes led to a significant reduction in colony formation and migratory abilities, as well as to an increase in tumor cell death. The results point to the combination of curcumin-loaded liposomes with PDT as a potentially useful tool for the treatment of papillomavirus-associated malignancies.

## 1. Introduction

Papillomaviruses belong to a group of tumor viruses associated with multiple cancers including cervical, anogenital, and a major subset of head and neck squamous cell carcinomas (HNSCC), which constitute about 4.5% of all solid tumors [1]. Cervical cancer is the fourth most frequent malignancy in women worldwide, accounting for 7.5% of all female cancer deaths [2,3], whereas HNSCC is ranked as the sixth most common form of cancer being responsible for 1–2% of all cancer deaths [4,5]. The standard regimen for the treatment of papillomavirus-associated cancers includes surgery, radiation, and chemotherapy. The side effects of surgical or radiation-based approaches can result in structural deformities, scars, hyperpigmentation, systemic side effects, and off-target destruction of normal tissues [6,7]. Additionally, the use of conventional therapies can induce multidrug resistance, resulting in treatment failure with recurrence of the disease [8]. To avoid toxicity and reduce side effects, alternative treatment strategies have been proposed. Photodynamic therapy (PDT) is one of the less invasive modalities that can be used instead of stressful conventional therapies. PDT is a minimally invasive therapeutic procedure in which nontoxic photosensitizers (PS) are administered systemically or applied locally, followed by their activation with light of specific wavelengths in the presence of cellular oxygen. The three mechanisms underlying PDT-mediated tumor destruction involve cell toxic reactive oxygen species (ROS) generation, tumor-associated vasculature damage, and immune response activation against the tumor. As a result, oxidation of cellular organelles can lead to apoptosis, necrosis, or autophagy of the cell [9,10]. The advantages of PDT include limited tissue damage, as illuminated light is restricted only to the photosensitized area with no major long-term systemic side effects [11]. Additionally, PDT does not interfere with chemo- and radiotherapy and, therefore, can be repeated as required. Curcumin, a natural polyphenolic compound extracted from rhizomes of curcuma longa, has demonstrated anti-inflammatory, antioxidative, and anticancer activities [12,13,14]. Its anticancer activity is based on multiple effects including inhibition of cancer growth, invasion, and metastasis [15]. Apart from these properties, curcumin has also been found to prevent multidrug resistance [16,17]. Moreover, a report from the National Cancer Institute (NCI) states that a curcumin formulation (Lipocurc^TM^, Signpath Pharma; Quakertown, PA, USA) has shown chemoprevention activity without any relevant toxicity in phase I clinical trials [18]. An oral dose of 8.0 g/day of curcumin is required for pharmacological efficacy, and various studies have demonstrated that no detectable toxicity was noted at doses of 10 g per day [19]. Curcumin in combination with irradiation (350–500 nm) showed significant antitumor activity resulting in curcumin also being recognized as a PS for PDT [20,21]. Curcumin possesses photobiological and photosensitizing activity. The fluorescence properties of curcumin can be utilized at an excitation wavelength of 457 nm for its maximum emission intensity [22]. It has been reported that the photobiological activity of curcumin was due to its excited state rather than due to photodegradation products of curcumin [23]. As a result of photoexcitation of curcumin, there is generation of singlet oxygens (^1^O_2_), which are highly reactive and toxic to cells, as well as ROS, leading to tumor destruction. Despite its numerous therapeutic effects, the clinical benefits of curcumin are limited due to poor solubility, rapid metabolism, and systemic elimination of the native compound [24]. These limitations can be overcome by using different drug carriers to improve curcumin solubility [25,26,27]. Among such drug carriers, liposomes have been broadly studied showing promising therapeutic effects [28,29]. Liposomes are artificially generated spherical vehicles containing one or more phospholipid bilayers and are considered as promising carrier systems for transporting both hydrophilic and hydrophobic drugs [30]. The bioavailability, stability, and circulation time of curcumin can be improved by encapsulation in liposomes [28]. PDT has been identified as a promising therapeutic tool for the treatment of papilloma virus-associated cervical and head and neck cancers [31,32]. The objective of this study was to evaluate the effects of PDT in combination with curcumin-loaded liposomes in papilloma virus-positive (PV+) cell lines. A set of three PV+ cell lines was selected. In addition to the HPV (human papillomavirus) -18 positive cervical cancer cell line HeLa and the HPV-16 positive HNSCC cell line UD-SCC-2, we included transiently growing cottontail rabbit papilloma virus (CRPV)-positive cells derived from the VX2 carcinoma of the New Zealand White (NZW) rabbit, which serves as an animal model for human HNSCC [33,34,35]. In this study, we used these cell lines to investigate the effects of PDT alone, curcumin liposomes only, and curcumin liposomes in combination with PDT on cell viability, apoptosis, cell proliferation, and migratory and colony formation abilities.

## 2. Results and Discussion

### 2.1. Physicochemical Properties of Curcumin Liposomes

**Figure 1:**
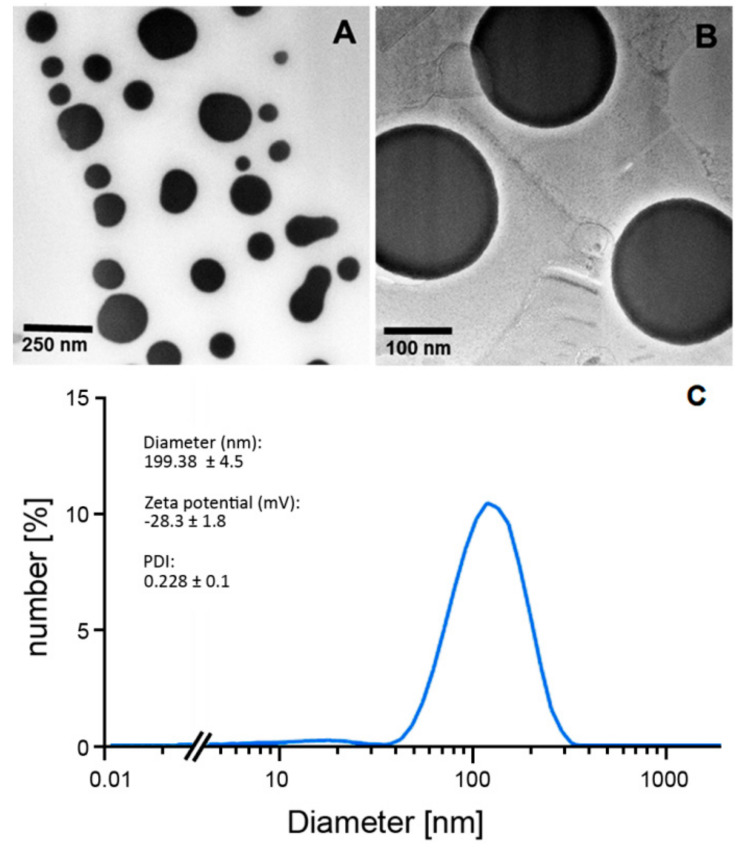
Physicochemical characteristics of curcumin liposomes. Transmission electron microscopical images depicting the morphology and size variance of curcumin liposomes (**A**,**B**) were generated as previously described by Duse et al. [29]. Shown are characteristic features of the used curcumin liposomes such as diameter (size) distribution, zeta potential, and polydispersity index (PDI) (**C**). ”//” in the x-axis shortens the logarithmic scale in the non-relevant lower range for better presentability of the actual size range of curcumin liposomes.

The stability, safety, and efficacy of drug delivery systems depend on various physical attributes such as particle size and the polydispersive index. The mean size distribution of liposomes was assessed by photon correlation spectroscopy (PCS) using a Zetasizer Nano ZS (Malvern Panalytical GmbH, Kassel, Germany). Using this method, the particle size (hydrodynamic diameter), zeta potential, and polydispersity index (PDI) can be determined. The data were expressed as the mean ± standard deviation derived from measurements of three independent experiments (Figure 1).

The dynamic light scattering analysis showed a PDI >0.2 suggesting the presence of a polydispersed liposome formulation [29]. The curcumin liposome formulation used here was as reported by Duse et al. In the study by Duse et al., the authors extensively described the generation and characterization of curcumin liposomes and particularly evaluated the effects of curcumin liposomes in conjunction with PDT on ROS generation, blood coagulation, hemolysis, and the microvasculature of the chicken chorioallantoic membrane. In addition, using the MTT (3-(4,5-dimethylthiazol-2-yl)-2,5-diphenyltetrazolium bromide) viability assay, the authors looked at cytotoxic effects in the human ovarian carcinoma cell line SK-OV-3 and in primary human coronary artery endothelial cells treated with curcumin-loaded liposomes together with PDT [29]. The present study builds on and extends the work of Duse et al. by, in addition to evaluating cellular viability, looking into the biological effects of curcumin-loaded liposomes and PDT on other tumor-associated features such as apoptosis, ability of cells to form colonies, and tumor cell migration. For this, three papillomavirus-associated cell lines were deployed since PDT was previously found to effectively treat HPV-positive cervical intraepithelial neoplasia (CIN) and vulvar HPV-16-positive cell lines [32,36]. In particular, we also included two papillomavirus-positive squamous cell carcinoma cell lines derived from the head and neck area since, in addition to cervical cancer of the uterus, head and neck cancer, specifically in the oropharyngeal region, is highly associated with HPV type 16.

### 2.2. Evaluation of Cell Viability after Dark Toxicity and Phototoxicity of Curcumin Liposomes

**Figure 2:**
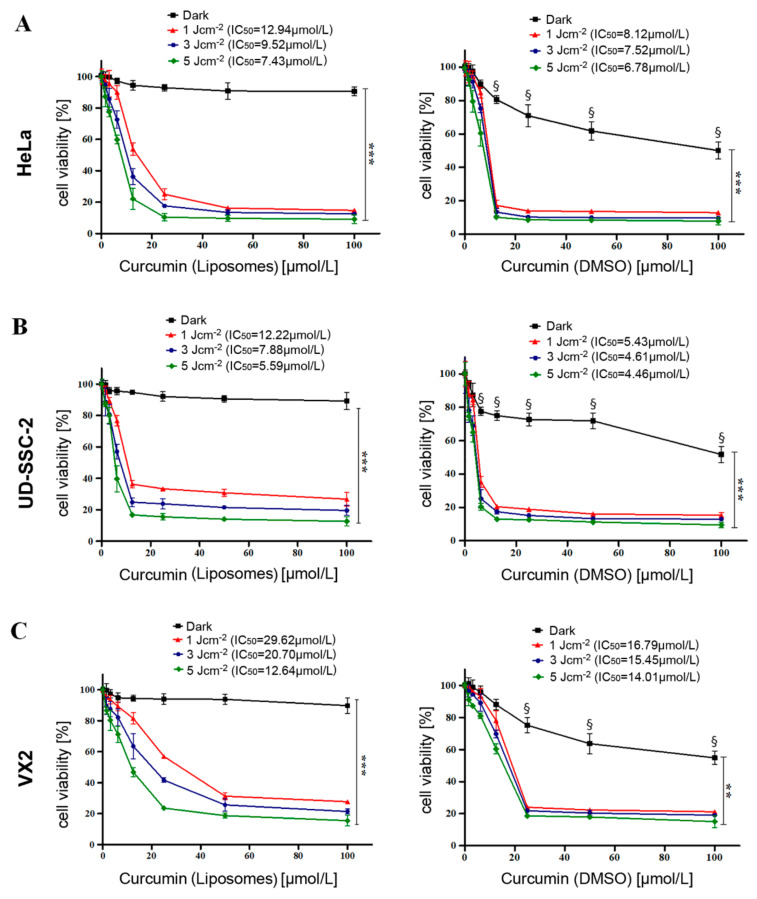
Evaluation of cellular viability in HeLa, UD-SCC-2, and VX2 cells. Cellular viability was assessed with the MTT (3-(4,5-dimethylthiazol-2-yl)-2,5-diphenyltetrazolium bromide) assay to determine the dark toxicity and phototoxicity of curcumin-loaded liposomes (**A, B, and C left**) and free curcumin using dimethyl sulfoxide (DMSO) as a vehicle (**A, B, and C right**). Dark toxicity and phototoxicity were determined by incubation of curcumin liposomes and free curcumin (in DMSO) for 4 h in the concentration range of 0–100 µmol/L at light fluences of 1, 3, and 5 J·cm^−2^. Dark refers to untreated cells (0 µmol/L curcumin) and cells treated with free curcumin (in DMSO) or curcumin liposomes without photodynamic therapy (PDT). The half maximal inhibitory concentration (IC_50_) values were calculated by nonlinear curve fitting for each light fluence. All samples were measured in triplicate, and the data are expressed as mean ± standard deviation. Statistical significances are indicated as *** *p* < 0.001, ** *p* < 0.01. § denotes that the viability of cells treated with free curcumin (in DMSO) is significantly (*p* < 0.001) lower than in the corresponding curcumin (liposomes)-treated cells.

MTT assays were performed to evaluate the dark toxicity (no light exposure) and phototoxicity of curcumin liposomes and free curcumin in HeLa, UD-SCC-2, and VX2 cells 24 h after exposure. This was carried out by incubation of cells with different concentrations (0–100 µmol/L) of curcurmin loaded liposomes and free curcumin dissolved in dimethyl sulfoxide (DMSO) for 4 h followed by subsequent exposure to different light fluences (1, 3, and 5 J·cm^−2^) using a prototype light-emitting diode (LED) device with blue light (wavelength λ = 457 nm). Initial incubation of all cells for 2 h did not show any significant difference in cellular viability (data not shown), indicating the need for increasing the incubation time. Treatment of all cell lines with free curcumin without irradiation showed a dose-dependent cellular toxicity referred to as dark toxicity (Figure 2A–C; right graphs). This dark toxicity, however, could also be due to the presence of DMSO serving as a vehicle of free curcumin. Contrary to this, curcumin-encapsulated liposomes did not show any significant toxicity in the dark (Figure 2A–C; left graphs), with all cell lines exhibiting 90–94% viability at maximal curcumin concentrations of 100 µmol/L. These data suggest that therapeutic toxicity can be abrogated by liposomal encapsulation and, thus, curcumin liposomes would remain nontoxic until illuminated by light of specific wavelengths. Irradiation alone resulted in no significant cell death in any of the cell lines. It was noticed in all cell lines that a combined treatment of curcumin liposomes and light irradiation (PDT) had a significant impact on cellular viability, resulting in light dose-dependent inhibition of cell proliferation as shown in Figure 2 (all graphs). The half maximal inhibitory concentration (IC_50_) value for each light dose was calculated by nonlinear curve fitting. A gradual reduction in half maximal inhibitory concentration of liposomes was noticed with increasing light fluence. The maximal phototoxicity was observed at a maximum radiation fluence of 5 J·cm^−2^. Since major effects were already seen at 3 J·cm^−2^, this light fluence was used for subsequent experiments. At the light fluence of 3 J·cm^−2^, the IC_50_ values of curcumin liposomes for HeLa, UD-SCC-2, and VX2 cells were 9.52 µmol/L, 7.88 µmol/L, and 20.70 µmol/L, respectively. The observed PDT effect after illumination was curcumin dose-dependent and highly significant (*p* < 0.001). These results are in accordance with previous reports pointing out a role of curcumin in the treatment of diverse maladies such as inflammatory diseases and cancer [19]. Similarly, López-Jornet et al. could demonstrate a synergistic effect of curcumin and ionizing irradiation on oral squamous cell carcinoma cells [20]. Feng and coworkers underlined the urgent need for specific curcumin formulations since the bioavailability of curcumin is very poor. The authors emphasized liposomal curcumin formulations as promising therapeutic vehicles [28]. Such curcumin-loaded tetraether liposomes showed prominent therapeutic efficacy, particularly when combined with PDT [29]. These observations are in agreement with a recent report using a curcumin nanoemulsion in conjunction with PDT on HPV-16 E6-transduced A431 cells [36].

### 2.3. Evaluation of Apoptosis as a Cause of Cell Death

**Figure 3:**
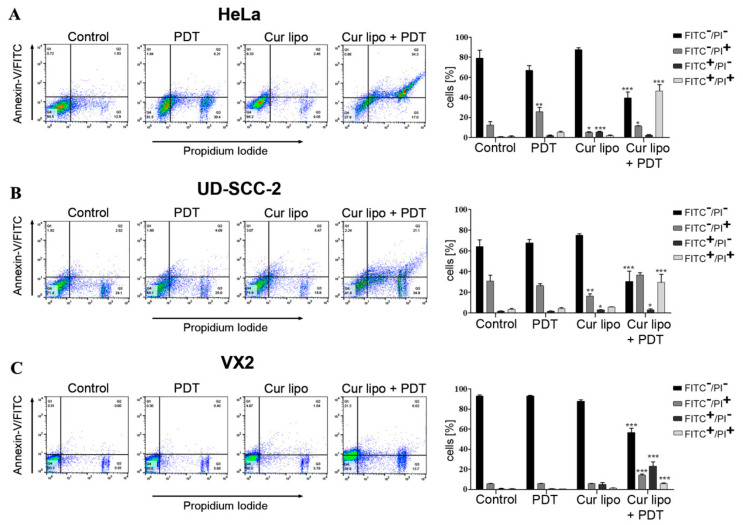
Evaluation of apoptosis as a cause of cell death in HeLa, UD-SCC-2, and VX2 cells after treatment with curcumin liposomes (Cur lipo) and photodynamic therapy (PDT). All cells were incubated with Cur lipo for 4 h and subsequently irradiated with a light fluence of 3 J·cm^−2^. After 24 h treatment, cells were co-stained with Annexin V-FITC (fluorescein isothiocyanate) and PI (propidium iodide). Representative flow cytometry micrographs are shown for HeLa (**A**), UD-SCC-2 (**B**), and VX2 (**C**) cells treated with PDT alone, Cur lipo alone, or a combination of Cur lipo with PDT. Untreated cells were used as a control. Q1 represents early apoptotic cells, Q2 represents late apoptotic or necrotic cells, Q3 represents necrotic cells, and Q4 represents live cells. Bar graphs represent the percentage of total apoptotic cells from at least three experiments. Data are shown as mean ± standard deviation, and statistical significances are indicated as *** *p* < 0.001, ** *p* < 0.01, * *p* < 0.05.

The underlying mechanism for inhibition of proliferation and cell death might be cellular apoptosis. Therefore, the flow cytometry-based Annexin V-FITC (fluorescein isothiocyanate)/PI (propidium iodide) assay was deployed to evaluate the rate of apoptosis in HeLa, UD-SCC-2, and VX2 cells after deploying various treatment modalities (mentioned in methods). Flow cytometry analysis of HeLa cells (Figure 3A) showed that combined treatment of curcumin liposomes and PDT exhibited an increase in the percentage (49.4% ± 6.0%) of Annexin V (FITC)-positive cells (consisting of early and late apoptotic or necrotic cells) when compared to cells irradiated with light only (7.4% ± 0.7%), cells incubated with curcumin liposomes only (7.4% ± 1.3%), and untreated (control) cells (1.8% ± 0.7%). Flow cytometry analysis of UD-SCC-2 cells (Figure 3B) showed that the combined treatment of curcumin liposomes and PDT exhibited an increase in the percentage (33.7% ± 6.7%) of Annexin V (FITC)-positive cells compared with cells irradiated with light only (5.8% ± 1.1%), cells incubated with curcumin liposomes only (8.7% ± 0.4%), and untreated cells (4.3% ± 0.5%). Similarly, flow cytometry micrographs and graphical data of VX2 cells (Figure 3C) showed that the combined treatment of curcumin liposomes and PDT on cells exhibited an increase in the percentage (29.0% ± 4.6%) of Annexin V (FITC)-positive cells when compared with cells irradiated with light only (1.1% ± 0.4%), cells incubated with curcumin liposomes only (6.5% ± 1.8%), and untreated cells (1.0% ± 0.2%). Flow cytometry results, therefore, demonstrate for all cell lines that curcumin liposomes in combination with PDT promote significant cell death. Since early apoptotic cells (Annexin V-FITC-positive and PI-negative) did not show a significant rise, apoptosis could not be unequivocally identified as the sole underlying cause of cell death in cells treated with curcumin liposomes and PDT.

### 2.4. Cell Viability Assessment via the SYTO9/PI Live/Dead Assay

**Figure 4:**
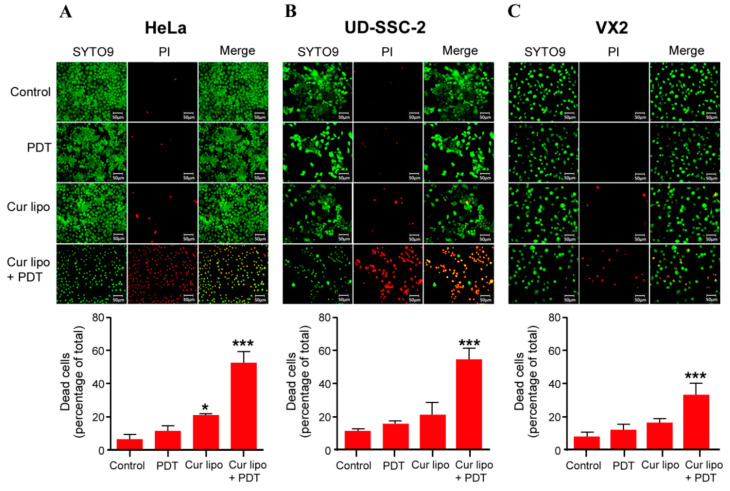
Live/dead staining of HeLa (**A**), UD-SCC-2 (**B**), and VX2 (**C**) cells. Cells were treated with photodynamic therapy (PDT) only, with curcumin liposomes (Cur lipo) only, or with Cur lipo in combination with PDT. Untreated cells were used as a control. All cells were stained with SYTO9 (green fluorescence) showing uniform green fluorescent nuclei, while propidium iodide (PI) only labeled nonviable or dead cells (red fluorescence) representing late apoptotic or necrotic cells. Bar graphs represent the quantification of dead cells (percentage) in the different treatment groups. Treated cells were compared to untreated control cells. The values are shown as mean ± standard deviation, and statistical significances are indicated as *** *p* < 0.001, * *p* < 0.05.

A live/dead assay was performed to differentiate between live and dead cells on the basis of cell membrane integrity [37]. HeLa (Figure 4A), UD-SCC-2 (Figure 4B), and VX2 (Figure 4C) cells were assayed using a dual staining procedure with SYTO9 and PI to determine the effects of light irradiation alone, as well as curcumin liposomes with and without light irradiation (PDT), on cellular viability. During staining, SYTO9 enters cells regardless of their membrane integrity and binds to DNA. After excitation, it emits a green fluorescence, while PI only enters cells with a disrupted membrane emitting a red fluorescence. Therefore, SYTO9 stains all cells, while PI stains necrotic and late apoptotic cells. Microscopic images of all cell lines demonstrate that cells without any treatment (control) and cells irradiated with light (PDT) only remained viable as indicated by the presence of green fluorescence. Cells treated with curcumin liposomes but without irradiation showed minor cell death (red fluorescence). In sharp contrast, treatment of cells with curcumin liposomes and PDT resulted in major cell death in all three cell lines. Interestingly, in a previous report, the authors used curcumin-loaded dextran micelles on C6 glioma cells. The authors observed major cell death when applying this formulation even in the absence of PDT [38] whereas in our study, using curcumin-loaded liposomes without PDT, we only observed minor cell death which markedly rose after applying PDT (Figure 4). This formulation, therefore, appears to be suitable for use in vivo since the toxic antitumor curcumin effect is activated only after PDT.

### 2.5. Influence of PDT Treatment on Colony Formation

**Figure 5:**
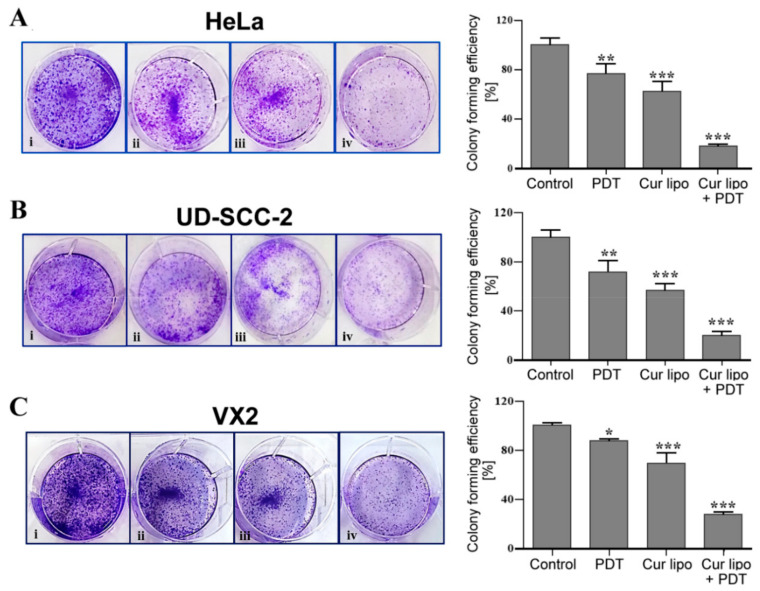
Colony formation assay with HeLa, UD-SCC-2, and VX2 cells. HeLa (**A**), UD-SCC-2 (**B**), and VX2 (**C**) cells were exposed to photodynamic therapy (PDT) only (**ii**), curcumin liposomes (Cur lipo) only (**iii**), or Cur lipo in combination with PDT (**iv**). Untreated cells were used as a control (**i**). Cell colonies of HeLa (**A**), UD-SCC-2 (**B**), and VX2 (**C**) cells were stained with 0.1% crystal violet dye. Graphical data represent the level (percentage) of colony formation after exposure to the various treatment modalities compared to control values. Values are presented as the mean ± standard deviation, and statistical significances are indicated as *** *p* < 0.001, ** *p* < 0.01, * *p* < 0.05.

The effects of various treatment strategies on HeLa, UD-SCC-2, and VX2 cell colony formation were evaluated by a colony formation assay (Figure 5). Cells without any treatment were considered as control. Colonies were evaluated 14 days after treatment. HeLa cells exhibited a dramatically reduced (18%) ability to form colonies in cells treated with curcumin liposomes and PDT in comparison to control cells. Cells treated with curcumin liposomes alone reached 63% and cells treated with only PDT reached 77% of the colony formation level observed for control cells (Figure 5A). In UD-SCC-2 cells, the colony formation ability was reduced to 21% after combined treatment with curcumin liposomes and PDT compared with untreated control cells, while cells treated with curcumin liposomes alone and cells treated with irradiation alone showed a colony formation ability of 57% and 72%, respectively (Figure 5B). VX2 cells treated with curcumin liposomes and PDT showed the lowest number (27%) of colonies in comparison to control cells. In contrast, cells treated with curcumin liposomes showed 69% and cells exposed to light irradiation alone showed 89% colony formation ability (Figure 5C). Therefore, colony formation assay results suggest that treatment of cell lines with a combination of curcumin liposomes and PDT could significantly reduce the number of colonies as compared to light irradiation only or curcumin liposomes alone.

### 2.6. Analysis of Cell Migration

**Figure 6:**
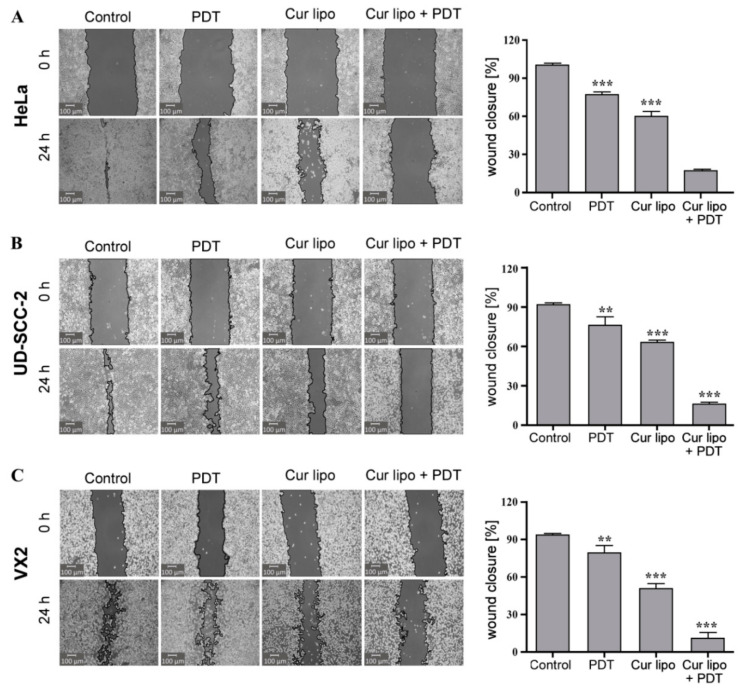
Cell migration (wound healing) assay in HeLa, UD-SCC-2, and VX2 cells. HeLa (**A**), UD-SCC-2 (**B**), and VX2 (**C**) tumor cells were treated with photodynamic therapy (PDT) only, curcumin liposomes (Cur lipo) only, or Cur lipo in combination with PDT. Images were captured at *t* = 0 h directly after scratching the cell layer and *t* = 24 h to evaluate scratch (wound) closure indicative of cellular migration. The cell-free area of the scratched region was measured with the Montpellier Ressources Imagerie (MRI) wound healing tool used with the ImageJ analysis software [41]. The level of cell migration is presented as the percentage of scratch (wound) closure observed 24 h after treatment compared to control values. Controls indicate untreated cells. The values are expressed as the mean ± standard deviation, and statistical significances are indicated as *** *p* < 0.001, ** *p* < 0.01.

A major cause of cancer morbidity and mortality is cancer metastasis, which is responsible for 90% of cancer deaths. The migration of cancer cells into the surrounding tissue is a primary step in tumor metastasis. Inhibition of cell migration can be a potential target to prevent cancer metastasis [39,40]. To investigate the influence of PDT on tumor metastasis, different treatment modalities were applied to HeLa, UD-SCC-2, and VX2 cells (Figure 6) followed by evaluation of tumor cell migration using the scratch (wound closure) assay. A significantly stronger inhibition of cell migration was observed microscopically in HeLa (Figure 6A), UD-SCC-2 (Figure 6B), and VX2 (Figure 6C) cells treated with a combination of curcumin liposomes and light irradiation. Respective graphs on the right depict the percentage of cells migrating into the scratched area, as observed at *t* = 24 h in HeLa (Figure 6A), UD-SCC-2 (Figure 6B), and VX2 (Figure 6C) cells, respectively. In HeLa cells, the percentages of cells migrating toward the scratched area in untreated control cells, and of cells treated with PDT only, curcumin liposomes only, and curcumin liposomes along with PDT were 99.0% ± 0.2%, 76.9% ± 2.1%, 59.5% ± 4.5%, and 16.9% ± 3.8%, respectively (Figure 6A). In UD-SCC-2 cells, the percentages of cells migrating toward the scratched area in untreated control cells, and of cells treated with PDT only, curcumin liposomes only, and curcumin liposomes along with PDT were 90.4% ± 0.8%, 71.2% ± 1.8%, 62.8% ± 2.2%, and 16.0% ± 2.3%, respectively (Figure 6B). In VX2 cells, the percentages of cells migrating toward the scratched area in untreated control cells, and of cells treated with PDT only, curcumin liposomes only, and curcumin liposomes along with PDT were 93.3% ± 0.7%, 79.0% ± 6.2%, 50.7% ± 4.3%, and 10.7% ± 4.5%, respectively (Figure 6C). Therefore, it becomes evident that curcumin liposomes photosensitized by light irradiation reduce cell migration and could contribute to the inhibition of tumor cell metastasis.

## 3. Materials and Methods

### 3.1. Reagents

Curcumin was purchased from Sigma Aldrich (Taufkirchen, Germany). 1,2-Distearoyl-*sn*-glycero-3-phosphocholine (DSPC) and 1,2-distearoyl-*sn*-glycero-3-phosphoglycerol (DSPG) were provided by Lipoid GmbH (Ludwigshafen, Germany). 3-(4,5-Dimethylthiazol-2-yl)-2,5-diphenyltetrazolium bromide (MTT) was purchased from Sigma Aldrich Chemie GmbH (Taufkirchen, Germany). Dimethyl sulfoxide (DMSO) was obtained from Carl Roth GmbH & Co. (Karlsruhe, Germany). Organic solvents including methanol (MeOH), ethanol (EtOH), and chloroform (CHCl_3_) were purchased from VWR International (Monroeville, PA, USA). Phosphate-buffered saline (PBS) pH 7.4 (with and without Ca^2+^/Mg^2+^) was freshly prepared, sterilized, and filtered using 0.2 µm polyethersulfone filters for all experiments.

### 3.2. Curcumin Liposomes: Preparation and Characterization

Curcumin liposomes were generated as described previously by Duse et al. [29] and are based on the thin-film hydration method [42]. Briefly, liposomes were formulated by mixing lipids (DSPC:DSPG; 80:20 molar ratio dissolved in chloroform/methanol 2:1; *v*/*v*) and curcumin (dissolved in methanol) in a round-bottom flask. Curcumin was added to the lipid mixture in a ratio of 1:30. The solvents were evaporated to obtain a thin film using a Laborota 4000 rotary evaporator (Heidolph Instruments, Schwabach, Germany) equipped with a vacuum pump (KNF Neuberger GmbH, Freiburg, Germany). Then, 20 mmol/L HEPES (4-(2-hydroxyethyl)-1-piperazineethanesulfonic acid) buffer solution (pH 7.4) was used to rehydrate the film followed by sonication in a bath-type sonicator (Elmasonic P30 H, Elma Schmidbauer GmbH, Singen, Germany) at 56 °C to obtain liposomal dispersion. The liposomes were then extruded using polycarbonate membrane filters (Nuclepore track-etch membrane, Whatman GmbH, Dassel, Germany) through 200 nm using an Avanti mini-extruder^®^ (Avanti Polar Lipids, Alabaster, AL, USA). The extruded liposomes were stored at 4 °C until further use. The hydrodynamic diameter and zeta potential of liposomes were measured by dynamic light scattering (DLS) and laser Doppler velocimetry (LDV), respectively, using a Zetasizer Nano ZS (Malvern Panalytical GmbH, Kassel, Germany) equipped with a 10 mW HeNe laser at λ = 633 nm with scattered light detection at an angle of 173°. Prior to the measurements, liposomes were diluted (1:100) with 20 mmol/L HEPES buffer (pH = 7.4) and placed in a clear disposable folded capillary cell (DTS1060, Malvern Panalytical GmbH, Kassel, Germany) for determining particle size and zeta potential. The curcumin concentrations were measured as previously described by Duse et al. [29]. The data were expressed as the mean ± standard deviation and were obtained from the measurements of three independent experiments.

### 3.3. Cell Culture

The cervical cancer-derived HeLa and the HNSCC-derived UD-SCC-2 (kindly provided by Prof. Dr. H. Bier, University of Düsseldorf, Germany) cell lines were grown in Dulbecco’s modified Eagle’s medium (DMEM) supplemented with 10% FBS (fetal bovine serum), 2 mmol/L l-glutamine (Capricorn Scientific GmbH, Germany), 50 μg/mL gentamicin (Biochrom GmbH, Germany), 100 U/mL penicillin/streptomycin (Capricorn Scientific GmbH, Germany), and 50 μg/mL amphotericin B (Biochrom GmbH, Germany). Fresh VX2 cells were derived from a VX2 carcinoma of a New Zeeland White (NZW) rabbit. The generation of VX2 tumors in NZW rabbits was approved by the regional board Giessen, Germany (V54-19c20-15 h01 MR 20/26 Nr. 83/2015) according to the German Animal Protection Law. Transiently growing VX2 cells (max. 150 passages) were then cultured in DMEM/Ham’s F-12 media containing 2 mmol/L l-glutamine (Capricorn Scientific GmbH, Germany) supplemented with 10% FBS, 50 μg/mL gentamicin (Biochrom GmbH, Germany), 100 U/mL penicillin/streptomycin (Capricorn Scientific, Germany), and 50 μg/mL amphotericin B (Biochrom GmbH, Germany). All three cell lines were cultured at 37 °C, 5% CO_2_ in a humidified incubator. Cells were grown as a monolayer until reaching approximately 80% confluency.

### 3.4. Photodynamic Therapy (PDT)

A low-power LED (light-emitting diode) device (Lumundus GmbH, Eisenach, Germany) was specifically designed to fit multiwell plates. The device was equipped with two different LEDs of 457 nm (blue) and 652 nm (red) wavelengths. It was supplied with the function to control settings for wavelength, current (mA) and irradiation time (s) as per the energy requirement. The device was able to deliver 220.2 W/m^2^ irradiance at a current of 100 mA and a wavelength of 457 nm (see also Figure S1, Supplementary Materials, for curcumin absorbance spectrum). The light dose (J·cm^−2^) supplied to the cells was equal to irradiance (W/cm^2^) multiplied by the irradiation time (s). For all PDT treatments, HeLa, UD-SCC-2, and VX2 cells were incubated with curcumin liposomes for 4 h at 37 °C, 5% CO_2_. Subsequently, cells were washed with PBS followed by addition of fresh media. Cells were then exposed to different light fluences and incubation was continued for 24 h. The cells were irradiated by light with a wavelength of 457 nm (blue) for 45, 136, and 227 s at a fluence of 220.2 W/m^2^ (100 mA) corresponding to 1, 3, and 5 J·cm^−2^, respectively, of the total light energy delivered.

### 3.5. Cell Viability and Irradiation Experiments

The MTT assay was deployed to assess the viability of cells treated with curcumin liposomes alone, PDT alone, or a combination of curcumin liposomes with PDT. Cells were seeded at ~1 × 10^4^ (HeLa and VX2) or ~6 × 10^4^ (UD-SCC-2) cells per well (0.35 cm^2^) in a 96-well transparent microtiter plate (Nunclon Delta, Thermo Fischer Scientific GmbH, Dreieich, Germany). Twenty-four hours after seeding, cells were incubated with curcumin loaded liposomes or free curcumin (dissolved in DMSO) at different concentrations ranging from 0–100 µmol/L for 4 h. Afterwards, cells were washed twice with PBS followed by addition of fresh medium. The cells were irradiated with light fluences of 1, 3, and 5 J·cm^−2^. The irradiated plates were incubated for 24 h in standard conditions (37 °C, 5% CO_2_). Nonirradiated plates (dark) were used as a control. After 24 h, MTT solution (final dilution in medium: 2 mg/mL) was added to the cells, and incubation was continued for another 4 h. Subsequently, the resulting formazan crystals were dissolved with DMSO, and the absorbance (Ab) was measured at 570 nm using the FLUOStar Optima plate reader (BMG Labtech, Ortenberg, Germany). Cell viability was calculated using the following formula:

Viability of cells (%) = ((Ab^sample^ − Ab^blank^)/(Ab^control^ − Ab^blank^)) × 100, (1)

where Ab^sample^ represents the absorbance of cells treated with curcumin liposomes or curcumin alone, and Ab^control^ refers to the absorbance of cells without any treatment. Ab^blank^ is the absorbance of wells containing media only.

### 3.6. Flow Cytometry of Annexin V-FITC/PI Stained Cells

Flow cytometry was performed to evaluate the underlying mechanisms of cell death using the Annexin V-FITC/PI kit (Annexin V-CFS, R&D Systems, Minneapolis, MN, USA). Briefly HeLa, UD-SCC-2, and VX2 cells were seeded in 6 well plates overnight at a density of 5–10 × 10^5^ cells per well. Upon reaching a confluency of 70–80%, cells were exposed to different treatments (cells treated with curcumin liposomes only, cells treated with light irradiation only, and cells treated with both curcumin liposomes and irradiation). Cells without any treatment were used as a control. Twenty-four hours after treatment, cells (1 × 10^5^) were harvested, washed in ice-cold PBS, and resuspended in 100 μL of (1×) binding buffer (1.4 mol/L NaCl, 0.1 mol/L HEPES (pH 7.4), 2.5 mmol/L CaCl_2_). The cells were incubated with 10 μL of fluorescein-conjugated Annexin V reagent for 15 min at room temperature in the dark. Thereafter, 400 μL of (1×) binding buffer containing 5 µg/mL PI, which stains late apoptotic and necrotic cells, was added to each tube and placed on ice. The sample measurements were performed using a BD LSR II flow cytometer (Becton Dickinson, Franklin Lakes, NJ, USA), and data analysis was performed with the FlowJo 10.6.2 software (Flowjo, LLC, Ashland, OR, USA). Early apoptotic, late apoptotic, and necrotic cells were estimated as the percentage of the total number of cells using the BD FACSDiva^TM^ software 7.0 (BD Biosciences, San Jose, CA, USA). Curcumin liposomes were incubated for 4 h, and light fluence was set to 3 J·cm^−2^ (IC_50_ at 3 J·cm^−2^: 9.52 µmol/L for HeLa, 7.88 µmol/L for UD-SCC-2, and 20.70 µmol/L for VX2 cells).

### 3.7. Live/Dead Staining Assay

The live/dead staining assay was performed on HeLa, UD-SCC-2, and VX2 cells to evaluate the toxicity of the different treatments (exposure of cells to curcumin liposomes only, exposure to light irradiation only, and exposure to a combination of curcumin liposomes and PDT). The cells were incubated with curcumin liposomes for 4 h followed by irradiation. After 24 h of treatment, cells were washed with PBS (containing Ca^2+^/Mg^2+^) and stained with a solution containing 2 µmol/L SYTO9 (S34854, Thermo Fisher Scientific, Waltham, MA, USA) and 4 µmol/L PI (propidium iodide, 81845, Sigma-Aldrich Co, St. Louis, MO, USA). The cells were incubated at 37 °C, 5% CO_2_ for 30 min, washed in PBS, and analyzed under a microscope (CKX53, Olympus America Inc., Center Valley, PA, USA). Curcumin liposomes were incubated for 4 h and light fluence was set to 3 J·cm^−2^ (IC_50_ at 3 J·cm^−2^: 9.52 µmol/L for HeLa, 7.88 µmol/L for UD-SCC-2, and 20.70 µmol/L for VX2 cells).

### 3.8. Clonogenic Survival Assay

A colony formation assay was performed to evaluate the ability of cells to proliferate after being exposed to the different treatment modalities [43]. HeLa, UD-SCC-2, and VX2 cells were plated in six-well plates at a density of 150 cells per well. Following different treatment procedures (PDT only, curcumin liposomes only, or curcumin liposomes with PDT), cells were incubated for two weeks at 37 °C and 5% CO_2_ without media change. After completing the 14 day incubation period, cells were washed with ice-cold PBS (containing Ca^2+^/Mg^2+^) and subsequently fixed with prechilled methanol for 10 min followed by another wash in PBS. Staining with 0.1% crystal violet dye was done to detect colony formation. Colonies with at least 50 cells were considered and counted. Curcumin liposomes were incubated for 4 h, and light fluence was set to 3 J·cm^−2^ (IC_50_ at 3 J·cm^−2^: 9.52 µmol/L for HeLa, 7.88 µmol/L for UD-SCC-2, and 20.70 µmol/L for VX2 cells).

### 3.9. Evaluating Cellular Migration

The scratch (wound closure) assay was deployed to analyze the migratory ability of cells after exposure to various treatment procedures [44]. HeLa, UD-SCC-2, and VX2 cells were seeded in six-well plates at a density of 5 × 10^5^ cells/well and cultured until cells reached 90% confluency. After cells were subjected to different treatments (PDT only, incubation of cells with curcumin liposomes in dark and in light), a linear scratch was made using a 10 µL sterile pipette tip followed by washing of cells with PBS and addition of fresh medium. The images were captured at *t* = 0 h to record the initial area of the scratch and at *t* = 24 h to evaluate effects of different treatments on the migratory ability of cells toward the scratch area. The images were photographed with the CKX53 microscope system (Olympus America Inc., Center Valley, PA, USA). ImageJ analysis software [41] (MRI_wound healing tool, written by V. Baecker; collaborators: N. Cahuzac and V. Georget; (c) 2010-2017, INSERM) was used to quantify the area of the scratch. The migration of cells toward the scratch area was expressed as the percentage of scratch cell migration (scratch cell migration (%) = ((Area*^t^* ^= 0 h^ − Area*^t^* ^= 24 h^)/(Area*^t^* ^= 0 h^)) × 100) [45]. Curcumin liposomes were incubated for 4 h and light fluence was set to 3 J·cm^−2^ (IC_50_ at 3 J·cm^−2^: 9.52 µmol/L for HeLa, 7.88 µmol/L for UD-SCC-2, and 20.70 µmol/L for VX2 cells).

### 3.10. Statistical Analysis

All experimental measurements were performed in triplicate, and the values were presented as the mean ± standard deviation. Nonlinear curve fitting functions were used to calculate the IC_50_ values obtained from the MTT assay. One-way ANOVA with post hoc test (Dunnett’s multiple comparison against control) was performed to identify statistically significant differences using the GraphPad Prism 5 software (San Diego, CA, USA). A *p*-value <0.05 was considered as statistically significant.

## 4. Conclusions

On the basis of the results from this study, it can be concluded that liposomal encapsulation allows for efficient delivery of curcumin to target cells. Curcumin liposomes were capable of generating a PDT-triggered response in three papilloma virus-associated tumor cell lines, leading to major cell death. It also became evident that phototoxic effects of curcumin liposomes in combination with PDT vary from one cell line to the other. This study revealed that papillomavirus-associated tumor cell lines treated with a combination of curcumin and PDT showed enhanced cell death, inhibition of cell growth, and reduction in colony formation and cell migration. These experimental findings point to PDT in combination with curcumin-loaded liposomes as a potential useful tool for the treatment of papilloma virus-associated tumors. Prospective in vivo studies are now required to evaluate the therapeutic benefit of curcumin-loaded liposomes in combination with PDT in the treatment of solid tumors such as HNSCC and cervical cancer.

## Supplementary Materials

The following are available online at https://www.mdpi.com/2072-6694/12/11/3278/s1, Figure S1. Absorption spectrum of curcumin.
