# Supplementary material for: Sensitivity of Papilloma Virus-Associated Cell Lines to Photodynamic Therapy with Curcumin-Loaded Liposomes
Source: Cancers (Basel). 2020 Nov 5;12(11):3278. doi: 10.3390/cancers12113278 (PMC7694491; doi:10.3390/cancers12113278)
Supplement: Supplementary file 1 [file cancers-12-03278-s001.pdf]

Supplementary Materials: Sensitivity of Papilloma Virus Associated Cell Lines to Photodynamic Therapy with Curcumin Loaded Liposomes

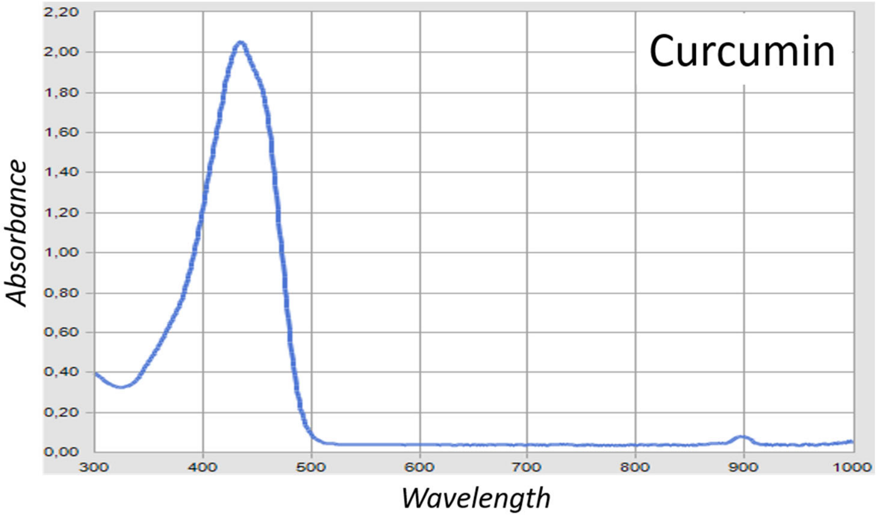

Figure S1. Absorption spectrum of curcumin.
